# xGDB: open-source computational infrastructure for the integrated evaluation and analysis of genome features

**DOI:** 10.1186/gb-2006-7-11-r111

**Published:** 2006-11-20

**Authors:** Shannon D Schlueter, Matthew D Wilkerson, Qunfeng Dong, Volker Brendel

**Affiliations:** 1 Department of Genetics, Development, and Cell Biology, Iowa State University, Ames, Iowa 50011-3260, USA; 2 Department of Agronomy, Purdue University, West Lafayette, Indiana 47907, USA; 3 Center for Genomics and Bioinformatics, Indiana University, Bloomington, Indiana 47405-3700, USA; 4 Department of Statistics, Iowa State University, Ames, Iowa 50011-3260, USA

## Abstract

The eXtensible Genome Data Broker (xGDB) provides a software infrastructure consisting of integrated tools for the storage, display, and analysis of genome features in their genomic context. Common features include gene structure annotations, spliced alignments, mapping of repetitive sequence, and microarray probes, but the software supports inclusion of any property that can be associated with a genomic location. The xGDB distribution and user support utilities are available online at the xGDB project website, http://xgdb.sourceforge.net/.

## Abstract 2

XGDB, a software infrastructure consisting of integrated tools for the storage, display and analysis of genome features (any property that can be associated with a genomic location, for example spliced alignments) in their genomics context is described.

## Rationale

Computational infrastructure is vital for all aspects of genome research. The assembled genomic sequence of an organism provides a natural scaffold for organizing biologic data. However, researchers are easily overwhelmed if they do not have the computational tools necessary to interpret the features of these assemblies [1-4]. Although a large number of useful tools are available, they exist primarily as *ad hoc *collections [5-7]. The xGDB software was designed to provide a framework for genomic data storage, display and analysis, and to provide integration of existing and novel genome analysis tools. The software is portable and easily installed for either public access or as a private workbench. It comes ready to use with the following features and capabilities: detailed feature record pages; detailed views of genomic contexts; support for online community annotation; utilities for storage of feature data in relational databases; effortless integration and attachment of analysis tools; transcript view, which is a novel nucleotide resolution view of genomic contexts; compressed storage and dynamic retrieval of feature evidence alignments; attachment and organization of multiple URLs to any feature in any context; and integrated heuristic searches based on feature identifier, alias, and/or description.

It is important to note that xGDB differs from and is complementary to database systems such as GMOD [8], EnsEMBL [9], and GenBank [10]. Unlike these systems, which are tasked to provide encompassing data storage, xGDB instances are applied to specific research oriented tasks, which are enabled by the browser and integrated analysis tools. Because of the varying reliability of genomic features, there is a strong need to go beyond simply plotting such features for display (as would be available in GBrowse [8], for example). Contextual analysis of genomic features often requires filtering each feature by criteria specific to an individual user's needs. Such filtering requires the development of a system around a genome browser that manages storage and display of the evidence that each feature is based on. Driven by this need, xGDB infrastructures provide interconnected analysis, visualization, and data management tools in a ready to use and easily extended package. The xGDB system is unique in providing this capability, for example integrating Geneseqer [11] spliced alignment features in plant-specific instances of xGDB.

An extensible infrastructure allows a wide array of data, tools, and analysis results to be brought together and provides the means by which to target their use in a focused manner. The xGDB package has been used to establish unique infrastructures tailored to the evaluation of genomic features. The xGDB instances available at PlantGDB [12] have been widely used in the analysis of genome annotation, gene structure determination, alternative splicing, and gene copy distribution [13-17]. Developing *ad hoc *methods for such analyses is expensive and time consuming. This cost is a major deterrent to many research endeavors and often leads to continuous redevelopment of analysis procedures [18-21]. Lack of stability leaves users questioning the accuracy of such analyses. The xGDB infrastructure provides both extensibility and procedural stability. Analysis procedures and results are made transparent to users, allowing them to formulate their own opinion of results and providing a means to reproduce and maintain each analysis.

In the following we first discuss the features and capabilities of an xGDB system as seen by end users. We then present the internal design and back-end components relevant to data providers and private installations. The installation is straightforward and requires basic knowledge of common open source software. For the purposes of illustration, we refer to AtGDB [22] and ZmGDB [23], which are publicly accessible xGDB instances established at PlantGDB. AtGDB and ZmGDB are based on the five assembled chromosomes of *Arabidopsis thaliana *and emerging genomic sequence assemblies of *Zea mays*, respectively. Additional plant genome xGDB systems are accessible through the PlantGDB website [24].

## Features and capabilities

The xGDB system is primarily accessed through dynamically generated web pages. These pages can be classified into context, record, and web service pages. Context pages present the location of genomic data sources in relation to surrounding features. Record pages localize pertinent external references, alignment results, and web service links. Web service pages allow a user to interact with data stored in the xGDB system, for example invoking BLAST for sequence comparisons [12,25] or GeneSeqer for spliced alignment of transcript sequences [11,26]. The whole set of web pages allows the system to quickly retrieve large amounts of data relevant to the user-specified task and control data presentation in a targeted and organized manner. By default, xGDB is configured to target data presentation for the purpose of evaluating gene structure annotation and genome annotation content, but xGDB has also been used to evaluate alternative splicing, microarray probe uniqueness, repetitive DNA positioning, and genetic marker placement.

### Viewing genomic regions in context

**Figure 1:**
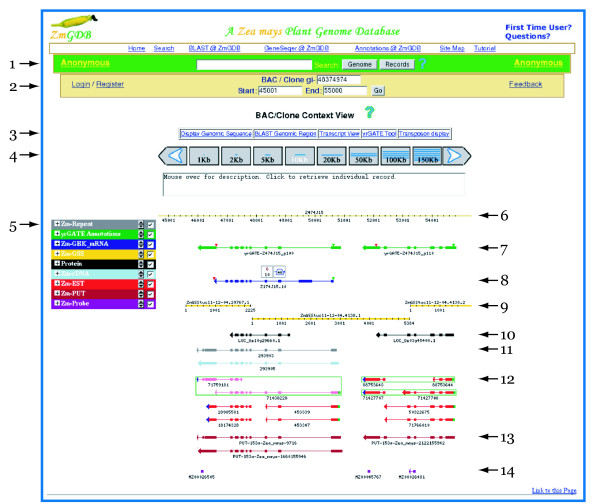
A ZmGDB context page focused on a *Zea mays *BAC assembly (accession Z474J15; GenBank id 48374974). A site header contains site navigation and search controls (items 1 and 2). Links to integrated webservices (item 3) and context navigation controls (item 4) are available. The feature control panel (item 5) and context graphic shows yrGATE community annotations (item 7), GenBank gene features (item 8), PlantGDB GSS assemblies (item 9), rice predicted protein alignments (item 10), cDNA alignments (item 11), EST alignments (item 12), PlantGDB Unique Transcript alignments (item 13), and MaizeArray microarray probe alignments (item 14) in the genomic region spanning bases 45,001 to 55,000 (item 6) of the assembled sequence. Exon features are displayed as filled rectangles connected by intronic features represented by similarly colored lines. Predicted start and stop codons of open reading frames are represented by green and red triangles, respectively. Arrowheads represent genomic strand orientation when this can be determined. Noncognate features are represented by alternative feature colors (pink for EST and grey for cDNA features). BAC, bacterial artificial chromosome; EST, expressed sequence tag; GSS, genome survey sequence.

On accessing an xGDB system, users are presented with navigational controls that allow them to search for genomic feature records and/or genomic locations. Navigational controls are displayed in a standard header at the top of all pages generated by the xGDB system (Figure 1 item 2). Depending on the configuration of xGDB, users may be presented with controls for selecting chromosomal coordinates from established genomic assemblies. These coordinates may be based on current or historic assembly versions, thus providing tracking of features that occurred in previous assemblies. In lieu of chromosome based navigation, controls for selecting individual coordinate locations in smaller assemblies such as a single bacterial artificial chromosome (BAC) or genome survey sequence (GSS) may be provided. These controls fetch the genomic region spanning the user supplied coordinates and display a genomic context page.

Genome context pages contain one or more sources of feature data such as curated gene annotations, locations of genomic markers, alignments of microarray probes, gene structure predictions, and alignments of expressed sequence tags (ESTs), cDNA, or assembled contigs of sequence. Figure 1 shows a context display of ZmGDB including community contributed gene annotations, GenBank documented gene feature annotations, GSS alignments, alignments of homologous proteins, cDNA and EST alignments, the alignment of PlantGDB Unique Transcript (PUT) assemblies, and the alignment of microarray probes (Figure 1 items 7 to 14). Features may be represented by an assortment of glyph colors and shapes that can be used to distinguish visually those properties that are specific to each. For example, in Figure 1 the context graphic showing EST alignment features (Figure 1 item 12) uses color to distinguish cognate alignments (shown in red) from those occurring due to the alignment of sequences from highly similar homologous loci (shown in pink). Additional glyph details provide indications of feature properties such as transcriptional strand (forward versus reverse), clonal orientation (5' versus 3'), corresponding clone pair sequences, annotated translational boundaries, and annotation incongruence.

From the context display, users can evaluate the level of alignment support for individual features as well as interrogate alternative features in the general vicinity. In the Figure 1 example, a researcher can ascertain that the structure of the *Zea mays *gene *TBP-2 *(shown in dark blue) as defined in the GenBank record of BAC accession Z474J15 (Figure 1 item 6) contains an unsupported exon. This conclusion is based on the alignment of cognate cDNA and EST alignments (Figure 1 items 11 and 12). Also, displayed are the alignments of homologous *Oryza sativa *protein annotations (Figure 1 item 10), two microarray probes (Figure 1 item 14), and three *Zea mays *GSS contigs (Figure 1 item 9) in the local vicinity of this gene annotation. A community contributed annotation (Figure 1 item 7, shown in green) documents one possible alternative transcript of this locus, as supported by EST and cDNA alignments. A second annotation documents the downstream locus as encoding a homolog to rice gene Os3g45400, which is adjacent to the rice TBP-2 gene on rice chromosome 3, thus identifying this region as microsyntenic between maize and rice.

Genome context pages provide navigational controls that allow users to pan, zoom, and customize their view while exploring the surrounding region. Preset buttons are available to zoom quickly to a desired nucleotide resolution (Figure 1 item 4). The track control panel (Figure 1 item 5) provides a legend of the available features and controls related to their display. Display options include positional controls for altering the vertical order in which features are displayed, a visibility control for hiding the display of feature groups, filters for viewing only cognate feature alignments, and selectors for viewing extensible glyph details such as those available with the GAEVAL extension discussed below. Adjusting the controls found in this panel will dynamically customize the genome context view without reloading the page.

**Figure 2:**
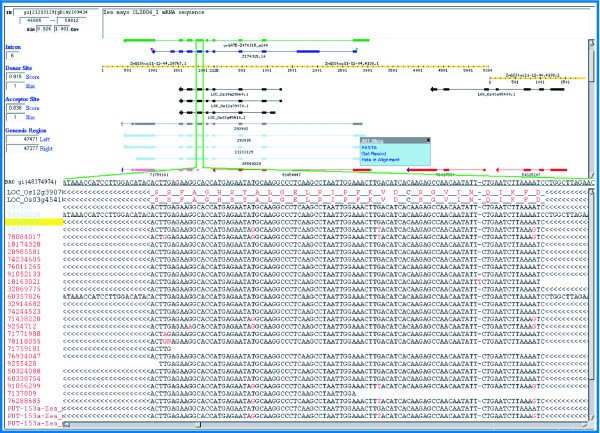
A ZmGDB transcript view context page associated with the genomic region depicted in Figure 1. The feature graphic in the top window pane is described in Figure 1. Information at the top and left of this pane is displayed when passing the cursor over feature elements. Currently displayed is the information associated with the sixth intron (immediately left of the green viewfinder) of the GeneSeqer spliced alignment of a *Zea mays *cDNA sequence (accession AV109414, GenBank id 21213129). The vertical green bars represent the view finder for the sequence view found in the bottom window pane. Red nucleotides shown in this view represent alignment mismatches with the genomic sequence.

Integrated web services related to the displayed genomic region are available via links (Figure 1 item 3), which are found above the context navigation controls. Typical services include display of the nucleotide sequence for the specified region, BLAST [25] query services, the yrGATE [27] community annotation tool, and a nucleotide level context page known as the transcript view. The transcript view context page displays detailed information about each feature as well as the nucleotide alignment of features derived from sequence alignment (Figure 2). Sequences of aligned features displayed in the transcript view sequence pane use the genomic region as a scaffold to present an inferred multiple sequence alignment. Differences between feature sequences and the genomic scaffold are displayed in red to ease detection of locus defining polymorphisms and single nucleotide polymorphisms. Coordinated scrolling of the sequence alignments and the sequence view indicator allow the transcript view to provide a viewing resolution suitable to detect genome sequence base calling errors, nearby alternative splice site usage, and other nucleotide level viewing requirements without numerous page reloads.

### Searching and browsing

The xGDB system provides intuitive and extensible search capabilities. Users may search for genomic locations or individual feature records using a variety of feature identifiers, aliases, keywords, or phrases entered into a common search control (Figure 1 item 1). Identifier searches are allowed to cascade through each feature component. Individual feature components provide an opportunity to modify the user supplied query to perform a heuristic search. For example, the official nomenclature [28] used to identify *Arabidopsis thaliana *gene annotations recommends identifiers of the form At2g42240.1. References to this gene annotation can be found at other databases under the identifiers AT2G42240.1, At2g42240, and AT2G42240. The heuristic search extensions found at AtGDB allow a user to locate this record by entering any of these identifiers.

**Figure 3:**
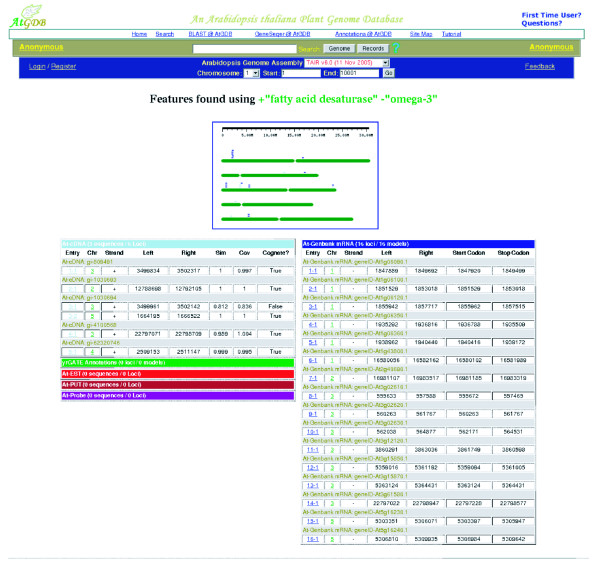
Search results at AtGDB using the query +"fatty acid desaturase" -"omega-3". The '+' and '-' operators represent inclusion and exclusion, respectively, following the convention of MySQL boolean text searches [39].

Descriptive searches based on keywords or phrases allow users to locate features of interest quickly. A user specified search that includes phrases enclosed by quotes or keyword inclusion/exclusion operators (+ and -, respectively), or that fails to locate a feature identifier triggers a descriptive search of available feature components. Searches resulting in multiple matching features will display a summary page detailing the matching features and their genomic locations. For example, Figure 3 shows the response to a request at AtGDB using +"fatty acid desaturase" -"omega-3". In this query, the exclusion phrase -"omega-3" allows a user to narrow the results of a typical descriptive query by removing results associated with omega-3, a common class of desaturase. As described above, feature components can be individually customized to provide extended search capabilities for descriptive searches.

### Evaluating feature records and their genomic alignment

Record pages provide information and web services pertinent to an individual feature. Users access record pages by clicking on a feature glyph from any context page (Figure 1 items 7 to 14) or using the record search control (Figure 1 item 1). Content modules, specific to each feature, control the display of record pages. These modules provide default record displays. Providers of xGDB resources have extensive control over the customization of these modules and may configure context page feature glyphs to link with record pages not generated by the xGDB system.

**Figure 4:**
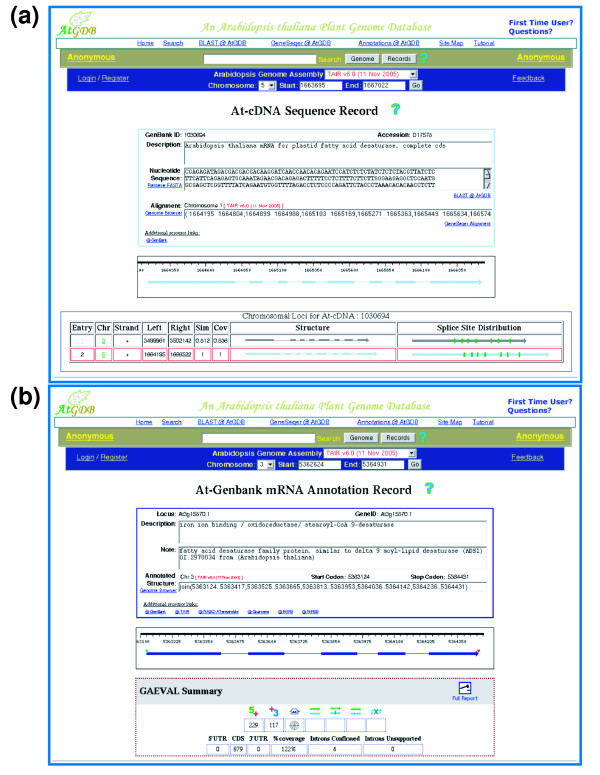
Individual feature pages found at AtGDB. **(a) **An AtGDB record page summarizing the GeneSeqer spliced alignment of an *Arabidopsis thaliana *cDNA sequence (accession BT020201, GenBank id 55733740). Feature structure glyphs found in the alignment loci summary table at the bottom of the window are as described in Figure 1. Green bars in the splice site distribution glyph represent the location of slice junctions in the processed mRNA transcript. **(b) **An AtGDB annotation record page detailing an *Arabidopsis *gene annotation (At3g15870.1). The GAEVAL Summary report at the bottom of the window displays information obtained using the integrated GAEVALxGDB services.

A typical record page includes information describing the feature source, peptide/nucleotide sequence(s), alignment coordinates, web service links, pertinent external website links, links to the alignment result on which the feature glyph is based, and tables summarizing the position and quality of the feature aligned to other genomic locations (Figure 4). Display of original alignment results is a key component of xGDB that allows users to evaluate the validity of individual features as well as the method used to generate their alignment. Collection of all alignment locations and quality measures of a feature in the loci summary table allows users quickly to determine homologous genomic locations and candidate overlapping genomic sequences. Display of structure and splice site distribution glyphs for these loci provide users with interesting details on the conservation of intron size and position.

### Packaged extensions

A major provision of the xGDB software design is extensibility of the core xGDB infrastructure. As such, extension of xGDB by adding third-party enhancements is encouraged. Two such enhancements, developed concurrently with xGDB, are the yrGATE gene annotation toolkit and the GAEVAL genome annotation evaluation toolkit. Both toolkits include fully functional standalone applications that can be incorporated into xGDB via web service extension modules.

The yrGATE toolkit provides an online portal for creation and submission of gene annotation. This web service is suitable for developing a large and nonexclusive community of annotators ranging in experience from professional curator to student. The yrGATE@xGDB extension module provides feature glyphs, search capabilities, context dependent web service links, and connections to evidence features stored in xGDB. This extension allows users to access yrGATE via web service links found on any context page for the purpose of creating an annotation. When xGDB is extended by this module additional navigational links are provided for all xGDB page headers. With these links, user can access the yrGATE annotation management pages that provide user account details, curation tools, and listings of accepted annotations.

The GAEVAL toolkit provides a system for the analysis of gene structure annotation by evaluation of supporting and incongruent evidence. This application is suitable for evaluating individual gene annotations by comparing both supporting and incongruent evidence. The GAEVAL@xGDB extension module enhances existing annotation feature components by adding glyph details to each feature, cuing users as to its GAEVAL evaluation. Glyph extensions include flags for exonic sequence coverage, splice site confirmation, and possible instances of alternative splicing, alternative transcriptional termination site usage, annotation fusion, annotation fission, or erroneous annotation overlap (Figure 1 item 8). This web service extension also provides additional record page details (Figure 4b) about each feature evaluation as well as links to GAEVAL query and report pages.

Combining these extensions under the xGDB infrastructure establishes a framework for targeting the efforts of would-be community annotators. Through access to the GAEVAL query service [29], lists of problematic annotations can be generated and sorted to provide a triage system for targeting annotators to interesting regions. The GAEVAL report service for each annotation can then be used to determine specific annotation alterations that are supported by current evidence. After manual evaluation of the proposed alterations, an annotator may use the yrGATE service [30] to provide an updated gene structure annotation. Upon acceptance of this user contributed annotation, the GAEVAL system is used to re-evaluate the current annotation, thereby documenting the presence of the new yrGATE submission.

## xGDB internals

We now describe the internal design and back-end components of xGDB accessible to data providers and users desiring private installations. We first present the overall system design, which is focused on modularity and extensibility. We then detail the feature component modules that are distributed with xGDB. Options for integrating alternative database structures and distributed database architecture are then discussed. Finally, we discuss options for installation and custom configuration of an xGDB system.

### Software design, modularity, and extensibility

The xGDB system consists of both user interface and data management components. Together, these components make xGDB highly modular and extensible. On the front end, the xGDB user interface is provided by a collection of CGI (common gateway interface) scripts. Core CGI scripts are maintained in data independent modules such that multiple xGDB systems may be operated using a single core installation. The AtGDB and ZmGDB systems illustrated herein, as well as all other species configurations maintained by PlantGDB, operate from a single xGDB core by taking advantage of this design feature. In addition, extended functionality such as that of the GAEVAL@xGDB service can be installed in a centralized location and made optionally accessible to all local xGDB systems.

**Figure 5:**
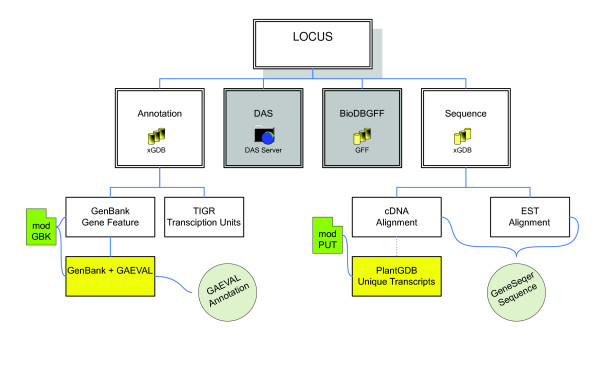
A partial representation of the object model for DSOs being used at AtGDB. Customized features derived from distribution objects are shown in yellow. Solid lines represent object inheritance. The dashed line connecting the PlantGDB Unique Transcripts feature represents instantiation of the cDNA DSO. Grey objects represent federated adaptors to external resources. DSO, data source object.

Data management and back-end database interoperability are provided by the xGDB database object and independent feature component modules discussed below. The use of modular feature components allows plug-in like inclusion of new feature sources as well as customization of existing sources. Feature components are built from an object oriented paradigm, in which required methods are gained through object inheritance and can be customized or extended by overriding individual method instances. These methods may take place in either the component class or individual instances of an existing class. Figure 5 depicts the object structure and point of customization of two features in use at AtGDB. The GenBank mRNA annotation feature uses a standard GenBank feature component that has been customized by addition of GAEVAL specific method instances. For this component, the underlying class itself was altered. The PlantGDB Unique Transcript feature, however, uses a standard cDNA feature component and is customized simply by addition of a modification file. This design allows for expansion and a variety of features to be uniquely represented with minimal additional effort.

### Feature component modules

Feature component modules consist of a Perl encoded DSO (data source object), web service scripts providing unique functionality to each feature component, data management scripts for loading features from flat files of various formats into a relational database management system, and supporting information necessary for feature configuration and customization. A variety of modules are available in the core xGDB distribution, including those encapsulating GenBank gene features, TIGR transcription units, and GeneSeqer expressed sequence spliced alignments. Incidentally, any genomic feature that can be positioned by a genomic coordinate can be developed into a feature component module. For example, with only minor modification of existing modules, we have added predicted repeats, GSS alignments, and microarray probe positions to the feature component modules in use at PlantGDB. As described in the following text, existing feature component modules and their common DSO design provides an ample infrastructure for managing most genomic features.

The DSO of each modular feature component inherits from a rich object framework that allows efficient method inheritance and less coding to develop objects encompassing new genomic feature sources (Figure 5). Currently, all DSOs descend from the Locus base object, which instantiates required object methods and provides a common object constructor. Most DSOs inherit the Locus object through hierarchical inheritance from second-tier objects such as the Annotation, Sequence, DAS (Distributed Annotation System), or BioDBGFF objects. These objects contribute standardized routines for searching, display, and interaction with feature components derived from each respective category. DSOs are often enhanced through multiple inheritance, as is the case with the cDNA and EST objects shown in Figure 5, which inherit both from the Sequence object and the GeneSeqerSequence object.

Method callbacks and subroutine hooks are used in the DSO framework to allow single instance customization of often modified object methods such as identifier and descriptive search routines, context region and record link publishers, and feature information HTML generators. The methods inherited from either the Annotation or Sequence objects encode subroutine hooks that allow a DSO to be customized by declaring a 'mod' file as an object configuration parameter. When declared, this 'mod' file is included in the DSO framework for its respective feature component. Although similar in function to Perl modules, a 'mod' file need not adhere to any packaging or naming conventions and is instantiated only when needed by an individual feature. In Figure 5, the GAEVAL enhanced GenBank gene feature DSO is shown to use a 'mod' file that provides an identifier validation routine responsible for heuristically altering a user supplied query to match feature identifier formats as found in the underlying MySQL database. The PUT (PlantGDB Unique Transcript) DSO also uses a 'mod' file. This modification is used to alter the cDNA DSO instance, thereby allowing it to encapsulate the PUT feature component.

### Integration with distributed and federated database systems

The xGDB database object manages the individual component features and provides adaptor methods for the relational database system of each component. Using an adaptor methodology, the choice of database management system, host, and scheme can be delegated to each feature component. As such, xGDB is capable of operating under distributed database architectures. One highly appealing use for such architecture is in maintaining an often changing feature set. For instance, local use of the individual EST and cDNA alignment feature available at AtGDB would necessitate a pipeline for continuous update as new sequences become available. This poses a challenge both in resource and time commitment for most small to moderately sized research groups. The ability of xGDB to utilize a distributed architecture, however, allows PlantGDB to provide direct connection to available PlantGDB feature sources (Table 1). Therefore, an individual xGDB maintainer need only configure their xGDB system to utilize this connection in order to remain up-to-date with the features found at PlantGDB.

The variety of genomic features, distribution sources, and distributed formats currently available for genomic context analysis necessitates an infrastructure system with federated data management capabilities. The modular design of xGDB allows creation of feature components specific to any distribution source or format. In addition to its native database architecture, the xGDB system is currently capable of using DAS [31] distribution sources and GFF (General Feature Format) databases [8] by providing feature component modules with federated data management adaptors. This allows integration with available tools and data distributed by projects such as Ensembl and GMOD. Examples and instructions for using these adaptors are provided with the xGDB installation notes.

### Installing and customizing xGDB

Setting up an xGDB system requires installation of the core xGDB distribution, installing an xGDB instance, populating a feature component module, and configuring the xGDB instance to include the feature component. Documentation and installation scripts are provided with the xGDB distribution to expedite this process. Instances are generally populated with multiple feature components. Components are associated with each xGDB instance through an instance configuration file. Additional xGDB instances can be configured for additional species or separation of publicly accessible resources from proprietary systems. Each subsequent instance may share the initial xGDB core and any feature components installed therein. Instance based customization of feature component modules as described above may be used to distinguish further individual xGDB resources.

Extensive options for customizing an xGDB instance are available. User interface properties such as color, image logos, and page layout are determined using a cascading style sheet. Modification of the default style sheet provided in the xGDB distribution allows an xGDB installer to quickly give any instance a unique look. Site navigation menus and controls can be customized using instance configuration files as well. These customization options are used with the xGDB instances at PlantGDB to provide additional informative content. This content includes species specific download pages; web pages relating relevant projects involving the use of xGDB, such as the characterization of U12-dependent introns using AtGDB; and links to relevant websites maintained by other research organizations. Third party groups and individuals are free to install, customize, and extend upon the xGDB system as provided for under the GNU general public license. In fact, one instance of xGDB has recently been applied to the annotation of *Glycine max *homeologous genomic sequences [32].

The xGDB distribution is available for download [33] and requires only widely available open source software. All distributed modules and required software run well on a variety of Unix based systems, including Linux and Macintosh OS X. The xGDB system performs well on server, desktop, and laptop computers. Utilizing the MySQL relational database manager, xGDB feature storage is currently limited only by the availability of feature data. For instance, the EST alignment feature available at AtGDB requires one-third or 1 Gb of disk storage and completes MySQL queries in approximately 1.5 s. Performance limitations are primarily dependent on the computer hardware xGDB is accessed from and the number of users accessing the system. In our own experience, the *Arabidopsis *and rice xGDB systems have been served from low-performance laptop computers to groups of 10 to 20 users with no noticeable performance loss, as well as from high-performance servers to the worldwide community. The xGDB systems interact with end-users through a combination of PHP and PERL generated web pages. Internet browsers that support HTML level 4, core JavaScript version 1.4 and higher, and Cascading Style Sheets level 2 and higher are required for complete user interface functionality. Default web pages have been design tested using Mozilla Firefox version 1.5.

## xGDB in summary

The xGDB system provides an infrastructure for organization of genomic data, analysis of a wide range of inquiries about such data, and online publishing of both data and analysis results. The extensible design of xGDB provides a packaged solution to many types of research applications. In particular, xGDB is well suited for small to moderately sized research groups desiring local access to genomic data or an out-of-the-box system for analyzing emerging data.

## xGDB software requirements

The xGDB system requires the following software packages: the Apache Web server [34], version 1.3 or higher; the PHP apache server API [35], version 3 or higher; and the Perl interpreter [36], version 5 or higher. In addition, it requires the following Perl modules found at CPAN [37]: DBI, DBD::mysql, GD, and CGI.

## xGDB support

The xGDB project is hosted on SourceForge.net, an online, open source development community. The complete xGDB distribution can be obtained from the xGDB project website [38]. This site includes utilities for user support, versioned distribution releases, bug reports, and feature requests. Forums at this site are regularly monitored by xGDB developers. The PlantGDB site also provides a user feedback utility to assist in user support for PlantGDB resources and requests. Links to this utility can be found in the header of all PlantGDB maintained web pages.

## Figures and Tables

**Table 1:** Feature sources provided by PlantGDB

Species	Genomic sequences			Annotations		Expressed sequences			
	
	Chr	BAC	GSS	GenBank	yrGATE	EST	cDNA	PUT	Probe
*A. thaliana*	5	-	-	34513	29	622,788	66,445	144,274	251,078
*B. rapa*	-	52	-	-	-	21,222	381	13,040	-
*G. max*	-	66	-	-	-	358,702	1,116	101,998	671,762
*L. esculentum*	-	89	-	467	-	199,873	3,291	40,966	112,528
*L. japonicus*	-	1374	-	170	-	149,878	224	43,592	-
*M. truncatula*	-	1644	-	18971	-	225,129	787	54,395	673,880
*O. sativa*	12	3462	-	68761	6	406,790	35,318	141,239	631,066
*P. trichocarpa*	-	173	-	-	-	89,943	119	29,640	-
*S. bicolor*	-	41	79,343	-	-	204,208	110	44,958	-
*T. aestvum*	-	57	-	-	-	853,621	2,386	243,326	-
*Z. mays*	-	2031	294,425	936	10	714,484	14,476	140,616	57,452

Column values represent the number of unique features/sequences made available at PlantGDB. The protein column represents the sum of all cross-species homologous protein alignments. Each expressed sequence may be responsible for multiple features by alignment to multiple loci. BAC, bacterial artificial chromosome; Chr, chromosome; EST, expressed sequence tag; GSS, genome survey sequence; PUT, PlantGDB Unique Transcript.
